# Supplementary material for: The interactome of intact mitochondria by cross-linking mass spectrometry provides evidence for coexisting respiratory supercomplexes
Source: Mol Cell Proteomics. 2017 Dec 8;17(2):216–32. doi: 10.1074/mcp.RA117.000470 (PMC5795388; doi:10.1074/mcp.RA117.000470)
Supplement: Supplemental Data [file supp_RA117.000470_133922_0_supp_23978_fzffwf.zip › spectra_annotation/mito_spectra_annotation/42-1-14-1-6-1.pdf]

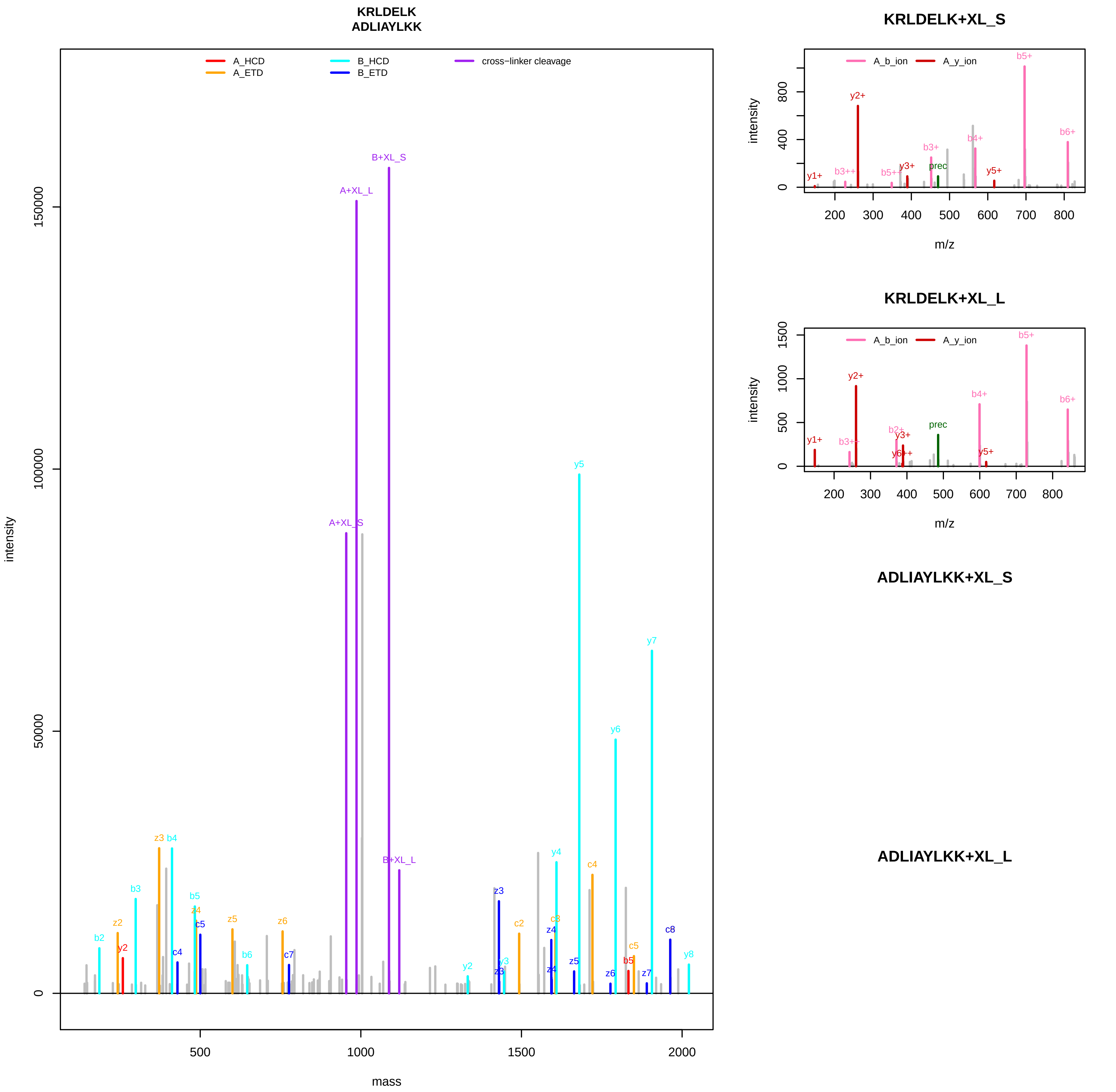

KRLDELK
ADLIAYLKK
A_HCD
A_ETD
B_HCD
B_ETD
cross−linker cleavage
intensity
mass
0
50000
100000
150000
500
1000
1500
2000
A+XL_L
B+XL_S
A+XL_S
B+XL_L
y5
y7
y6
y4
c4
z3
c3
c2
z4
z5
z6
b5
c5
z7
c8
y8
y2
y3
b2
b3
b4
b5
b6
z2
y2
z3
z4
z5
z6
c4
c5
c7
KRLDELK+XL_S
A_b_ion
A_y_ion
intensity
m/z
0
400
800
200
300
400
500
600
700
800
y1+
b3++
y2+
b5++
y3+
b3+
prec
b4+
y5+
b5+
b6+
KRLDELK+XL_L
A_b_ion
A_y_ion
intensity
m/z
0
500
1000
1500
200
300
400
500
600
700
800
y1+
b3++
y2+
b2+
y3+
y6++
prec
b4+
y5+
b5+
b6+
ADLIAYLKK+XL_S
ADLIAYLKK+XL_L
